# Supplementary material for: Rational structure-guided design of a blood stage malaria vaccine immunogen presenting a single epitope from PfRH5
Source: EMBO Mol Med. 2024 Sep 2;16(10):2539–59. doi: 10.1038/s44321-024-00123-0 (PMC11473951; doi:10.1038/s44321-024-00123-0)
Supplement: Supplementary file 1 — Table EV1 [file 44321_2024_123_MOESM1_ESM.docx]

***Table EV1: interactions list***

The left-hand column shows the PfRH5 residue number. The second column shows the equivalent residue in the RH5-34EM immunogen, with residues shown in green boxes being those which have their identity retained from PfRH5. The remaining columns show the interactions observed in crystal structures of antibody and either PfRH5 or RH5-34EM, with residues which make interactions in green and those which do not in red.

| Residue in PfRH5 | Residue in RH5-34EM | 9AD4 | R5.016 | | R5.034 | |
| --- | --- | --- | --- | --- | --- | --- |
|  |  | PfRH5 | PfRH5 | RH5-34EM | PfRH5 | RH5-34EM |
| Y200 | K46 | **-** | **-** | **+** | **-** | **-** |
| G201 | K47 | **-** | **+** | **+** | **-** | **-** |
| K202 | K48 | **+** | **+** | **+** | **-** | **-** |
| Y203 | K49 | **-** | **-** | **-** | **-** | **-** |
| I204 | I50 | **-** | **+** | **+** | **-** | **-** |
| A205 | A51 | **+** | **+** | **+** | **-** | **-** |
| D207 | E53 | **-** | **+** | **-** | **-** | **-** |
| A208 | A54 | **-** | **+** | **+** | **+** | **+** |
| F209 | F55 | **+** | **+** | **+** | **+** | **+** |
| K211 | K57 | **-** | **+** | **+** | **-** | **-** |
| K212 | K58 | **+** | **+** | **+** | **+** | **+** |
| I213 | I59 | **+** | **-** | **-** | **-** | **-** |
| E215 | E61 | **-** | **+** | **+** | **-** | **-** |
| A216 | A62 | **-** | **-** | **-** | **-** | **-** |
| D218 | D64 | **-** | **-** | **-** | **-** | **-** |
| K219 | K65 | **-** | **+** | **-** | **+** | **+** |
| V220 | V66 | **-** | **-** | **-** | **-** | **-** |
| N323 | E15 | **-** | **-** | **-** | **+** | **-** |
| K327 | K19 | **-** | **+** | **+** | **+** | **+** |
| I328 | I20 | **-** | **-** | **-** | **-** | **-** |
| M330 | M22 | **-** | **-** | **-** | **+** | **+** |
| D331 | D23 | **+** | **+** | **-** | **+** | **+** |
| K333 | K25 | **-** | **-** | **-** | **-** | **-** |
| N334 | N26 | **+** | **-** | **-** | **+** | **+** |
| Y335 | Y27 | **+** | **+** | **+** | **-** | **-** |
| T337 | T29 | **-** | **-** | **-** | **-** | **-** |
| N338 | N30 | **+** | **-** | **+** | **+** | **+** |
| L339 | L31 | **+** | **+** | **+** | **-** | **-** |
| E341 | E33 | **+** | **-** | **-** | **-** | **-** |
| Q342 | Q34 | **+** | **-** | **-** | **-** | **+** |
| S344 | A35 | **+** | **-** | **-** | **-** | **-** |
| C345 | N36 | **-** | **-** | **+** | **-** | **-** |
