# Supplementary material for: Rational structure-guided design of a blood stage malaria vaccine immunogen presenting a single epitope from PfRH5
Source: EMBO Mol Med. 2024 Sep 2;16(10):2539–59. doi: 10.1038/s44321-024-00123-0 (PMC11473951; doi:10.1038/s44321-024-00123-0)
Supplement: Supplementary file 2 — Table EV2 [file 44321_2024_123_MOESM2_ESM.docx]

***Table EV2: Sequences of designed immunogens***

Protein sequences for the designed immunogens. The residues labelled red in 3A, 3B and 3C are the cysteine residues added to introduce disulphide bonds.

| Immunogen | Sequence |
| --- | --- |
| 1 | GSGSYQDVLQKAEEKLRKIEMDAKNYRTNLEEQKDELSKTDDEKDKKKIAIEAFIKKIKEAADKVAREAEEELRKLKDKNQVDSQELDKAEDKAKKKADELKDKIDNIEDDARKWLDQ |
| 2 | GSGSAEDVLKEAEEKLRKIAMDAKNYRTNLEEQKQELNKTDEERERKRIAIEAFIKKIEEAADKVAREAKDKLDDLKKKNQVDEKKLEEVKQKVEREAREARRIIREAKDDAEKWLKQ |
| 3 | GSGSYQDVARKAKEKLDKIEMDAKNYETNLKEQANNADKTEEYRKKKKIAIEAFLKKIEEAADKVAREAKQRLDELEKKNQVDKEELEKAKEEVEKRARELRRRIREILERAKKWLDQ |
| 4 | GSGSYQDVAREAEEKLRKIEMDAKNYATNLEEQRDELSKTEEEIKKKKIAIEAFIKKIAEAADKVAREAEEELEKLKRKNQVDSKRLEDAKKRVKKLAEELKERIERIREKAEKWLKQ |
| 5 | GSGSYQDVAERAERKLRKIEMDAKNYRTNLEEQKDELAKTEEEIKKKKIAIEAFIKKIKEAADKVAREADRELDELKKKNQVDSEELEKAKDKVRKWAEELRRRIDEAKKDAEKWLKQ |
| 6 | GSGSYQDVKKEAEEKLDKIEMDAKNYRTNLEEQRQQLAKTEEEIKKKKIAIEAFIKKIEEAADKVAREAEEKLDRLKKKNQVDEKKLEEAKDDVKDKADEVRKKIRDAKDDAEKWLKQ |
| 7 | GSGSYQDVAREAKERLEKIEMDAKNYRTNLEEQKETLSKTEEEIKKKKIAIEAFIKKIEEAADKVAREAEERLRELEKKNQVDKNKLEKAEDEVKKKADEVRDKIRNARDDAEKWLKQ |
| 8 | GSGSYQDVAREAKERLDKIEMDAKNYRTNLEEQKDELSKTEEEIKKKKIAIEAFIKKIKEAADKVAREAKKRLDELKKKNQVDSEKLDKAKEEVEKKARELKKKIEEIREDAEKWLKQ |
| 9 | GSGSYQDVAREAKEKLEKIEMDAKNYRTNLEEQRDQLAKTQEEIQKKKIAIEAFIKKIEEAADKVAREADDKLDDLKKKNQVDSQELDKAKDEVRKKAEELKKKIREAREDAEKWLKQ |
| 3A | GSGSYQDVCRKAKEKLDKIEMDAKNYETNLKEQANNADKTEEYRKKKKIAIEAFLKKIEEAADKVAREAKQRLDELEKKNQVDKEELEKCKEEVEKRARELRRRIREILERAKKWLDQ |
| 3B | GSGSYQDVARKAKEKLDKIEMDAKNYETNLKEQANNADKTEEYCKKKKIAIEAFLKKIEEAADKVAREAKQRLDELEKKNQVDKEELEKAKEEVEKRARELRRRIREILERAKKWCDQ |
| 3C | GSGSYQDVCRKAKEKLDKIEMDAKNYETNLKEQANNADKTEEYCKKKKIAIEAFLKKIEEAADKVAREAKQRLDELEKKNQVDKEELEKCKEEVEKRARELRRRIREILERAKKWCDQ |
