# Supplementary material for: Rational structure-guided design of a blood stage malaria vaccine immunogen presenting a single epitope from PfRH5
Source: EMBO Mol Med. 2024 Sep 2;16(10):2539–59. doi: 10.1038/s44321-024-00123-0 (PMC11473951; doi:10.1038/s44321-024-00123-0)
Supplement: Supplementary file 3 — Table EV3 [file 44321_2024_123_MOESM3_ESM.docx]

***Table EV3: Crystallographic statistics***

|  | RH5-34EM: R5.016 | PfRH5:R5.034 | RH5-34EM:  R5.034 |
| --- | --- | --- | --- |
| **Data collection** |  |  |  |
| Space group | - P2_1_ 2_1_ 2_1_ | - P2_1_ 2_1_ 2_1_ | - P6_1_ |
| Cell dimensions |  |  |  |
| a, b, c (Å) | 64.281, 114.01, 217.09 | 82.75, 82.99, 118.75 | 111.83, 111.83, 91.57 |
| α, β, γ (°) | 90, 90, 90 | 90, 90, 90 | 90, 90, 120 |
| Wavelength | 0.9795 Å | 0.9786 Å | 0.9999 Å |
| Resolution (Å) | 217.10 – 1.63 (1.66 – 1.63) | 82.99-2.40  (2.49-2.40) | 55.92-1.75  (1.78-1.75) |
| Total Observations | 1306445 (64462) | 420026 (44946) | 655283 (30833) |
| Total Unique | 199219 (9819) | 32741 (3402) | 65543 (3252) |
| *R*_pim_ (%) | 3.3 (88.9) | 3.6 (46.4) | 4.4 (119.7) |
| *CC_1/2_* | 1.00 (0.52) | 0.999 (0.911) | 0.998 (0.598) |
| *I/σ(I)* | 12.0 (1.3) | 15.2 (2.0) | 9.6 (0.9) |
| Completeness (%) | 100.0 (100.0) | 100.0 (100.0) | 100.0 (99.9) |
| Multiplicity | 6.6 (6.6) | 12.8 (13.2) | 10.0 (9.5) |
| Wilson B factor | 25.0 | 55.49 | 25.22 |

| **Refinement** |  |  |  |
| --- | --- | --- | --- |
| Reflections | 199083 | 32667 | 65500 |
| *R*_work_ / *R*_free_ (%) | 19.10/20.59 | 25.74/30.46 | 19.86/21.84 |
| Average B factor | 36.0 | 72.0 | 44.0 |
| Number of residues |  |  |  |
| Protein | 1392 | 708 | 667 |
| Glycerol | 4 | 0 | 0 |
| Water | 1232 | 65 | 419 |
| R.m.s deviations |  |  |  |
| Bond lengths (Å) | 0.008 | 0.006 | 0.009 |
| Bond angles (°) | 0.92 | 0.87 | 0.95 |
| Ramachandran plot |  |  |  |
| Favored (%) | 99.4 | 96.4 | 97.5 |
| Allowed (%) | 0.6 | 3.6 | 2.5 |
| Outliers (%) | 0 | 0 | 0 |
