# Supplementary material for: Rational structure-guided design of a blood stage malaria vaccine immunogen presenting a single epitope from PfRH5
Source: EMBO Mol Med. 2024 Sep 2;16(10):2539–59. doi: 10.1038/s44321-024-00123-0 (PMC11473951; doi:10.1038/s44321-024-00123-0)
Supplement: Supplementary file 4 — Table EV4 [file 44321_2024_123_MOESM4_ESM.docx]

***Table EV4: binding parameters derived from surface plasmon resonance***

| Interaction | k_a_ (1/Ms) | k_d_ (1/s) | K_D_ (nM) | Chi^2^ |
| --- | --- | --- | --- | --- |
| RH5-34EM vs 9AD4 | 6.49 x 10^6^ | 0.02206 | 3.4 | 0.706 |
| PfRH5 vs 9AD4 | 3.65 x 10^5^ | 5.75 x 10^-4^ | 1.58 | 3.97 |
| RH5-34EM vs R5.016 | 4.78 x 10^5^ | 0.05482 | 115 | 4.09 |
| PfRH5 vs R5.016 | 2.08 x 10^5^ | 4.95 x 10^-4^ | 2.38 | 4.63 |
| RH5-34EM vs R5.034 | 1.11 x 10^7^ | 8.07 x 10^-4^ | 0.073 | 10.6 |
| PfRH5 vs R5.034 | 2.89 x 10^5^ | 2.70 x 10^-5^ | 0.093 | 1.51 |
