# Supplementary material for: Rational structure-guided design of a blood stage malaria vaccine immunogen presenting a single epitope from PfRH5
Source: EMBO Mol Med. 2024 Sep 2;16(10):2539–59. doi: 10.1038/s44321-024-00123-0 (PMC11473951; doi:10.1038/s44321-024-00123-0)
Supplement: Supplementary file 10 — Expanded View Figures [file 44321_2024_123_MOESM10_ESM.pdf]

Expanded View Figures

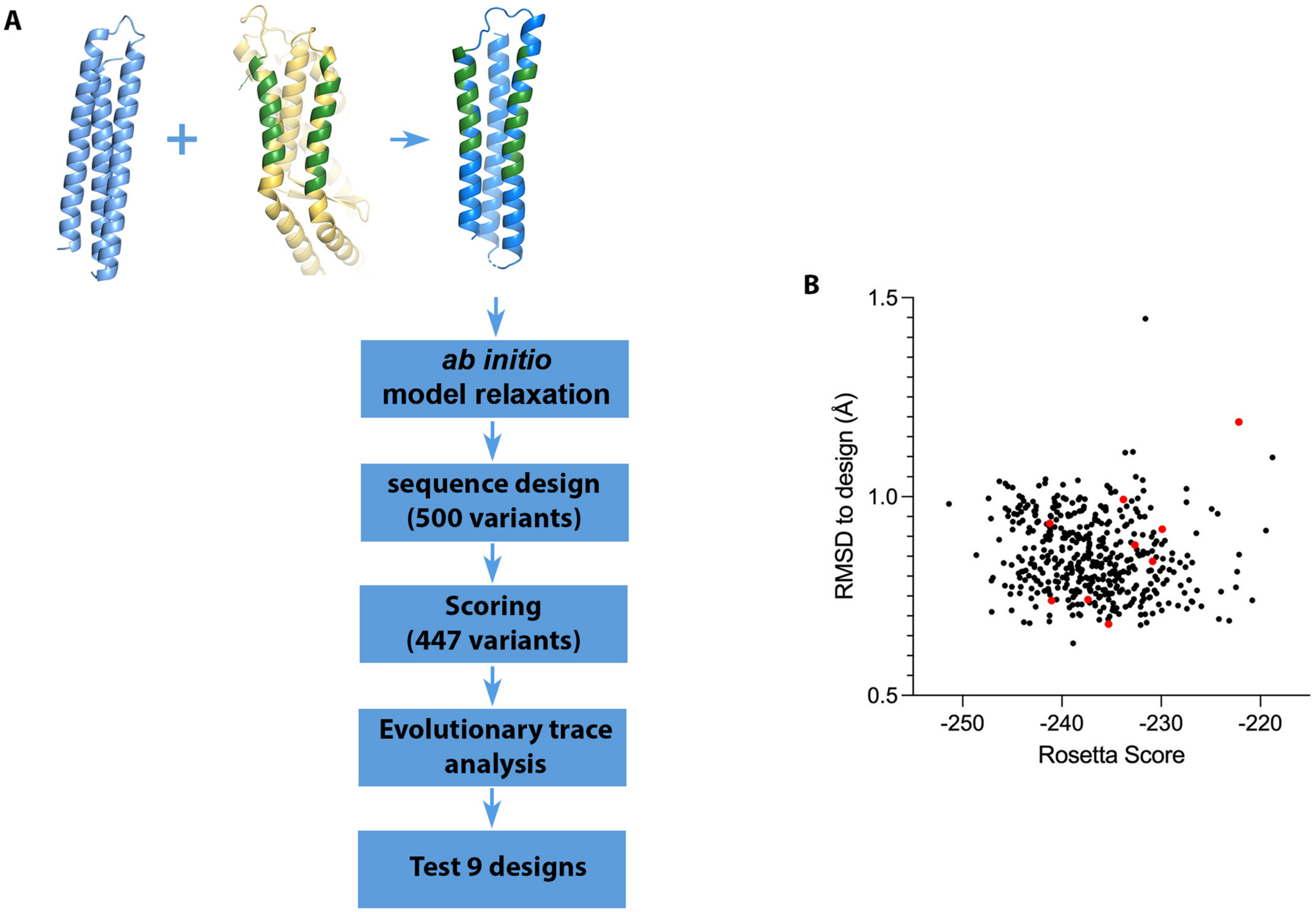

Figure EV1. Design process.

(**A**) A schematic showing the design of RH5-34EM. The 9AD4 epitope (green) of PfRH5 (yellow) was grafted onto a synthetic three-helical bundle scaffold (blue), generating an initial design, followed by a Rosetta-based design strategy. (**B**) A plot of Rosetta score against predicted root-mean square deviation to the design of the 447 sequence variants passing the scoring criteria. The nine red circles are for the designs taken forward for testing (designs 1–9).

 © The Author(s)

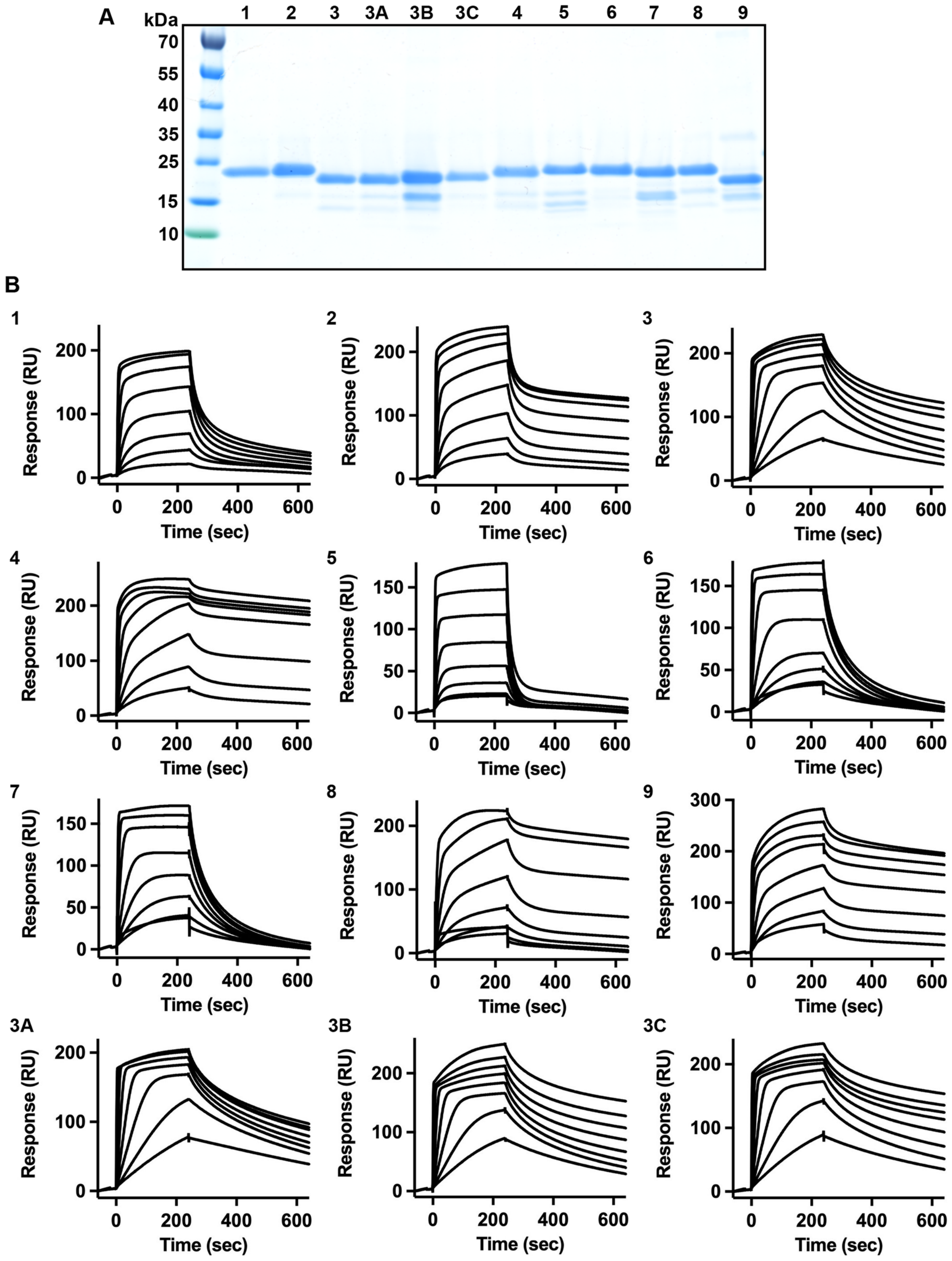

Figure EV2. Characterisation of epitope mimic designs.

(**A**) SDS PAGE gel for the twelve designs, stained with Coomassie. The designs vary in molecular weight from 16.2 to 16.5 kDa. (**B**) Surface Plasmon Resonance traces for the twelve designs. In each case, antibody 9AD4 was captured on the chip surface and a dilution series of each epitope mimic, from a maximum concentration of 500 nM, was flowed over this surface.

© The Author(s)

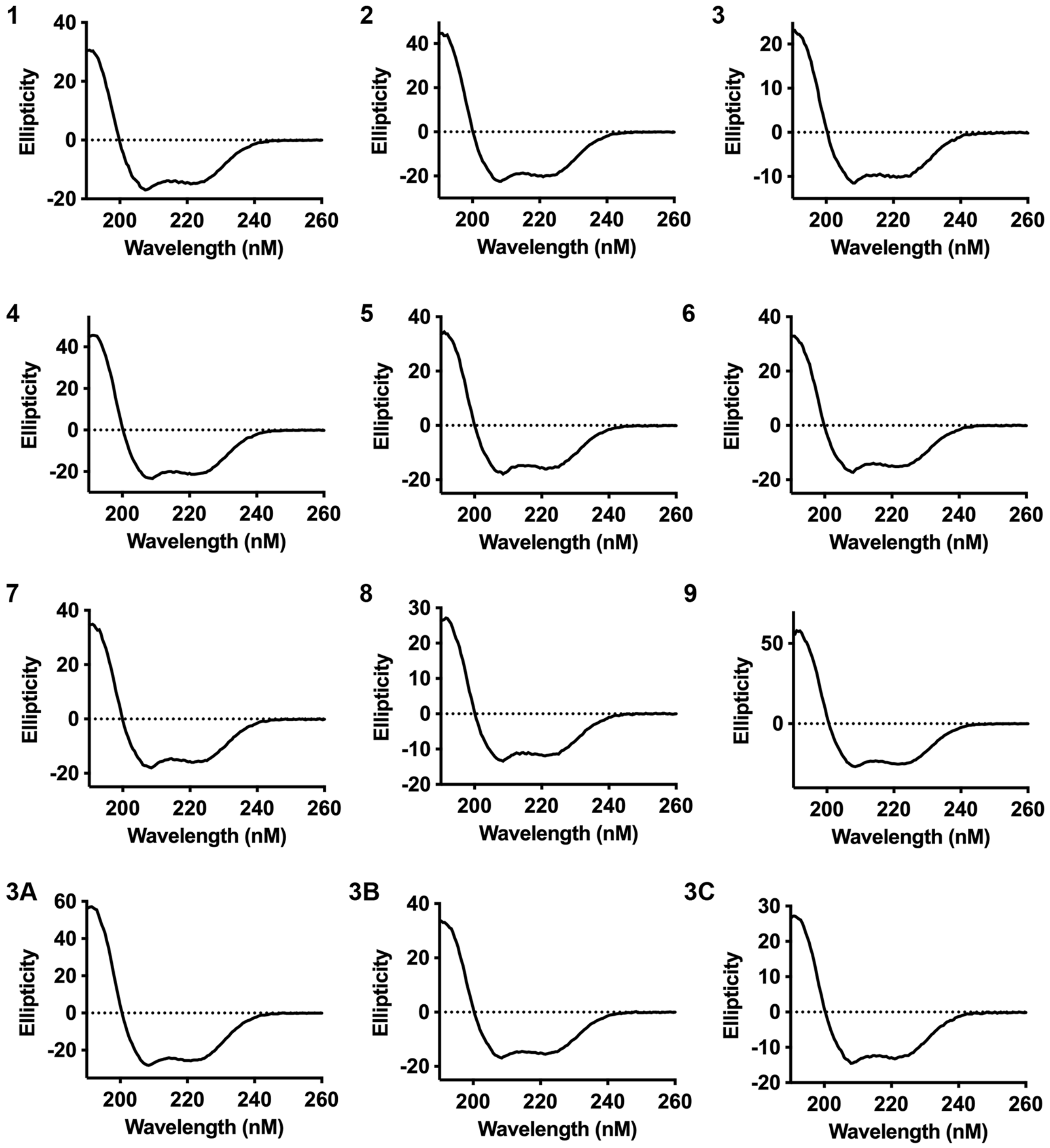

Figure EV3. Circular dichroism traces for the twelve designs.

Circular dichroism measurements for the twelve different epitope mimic designs.

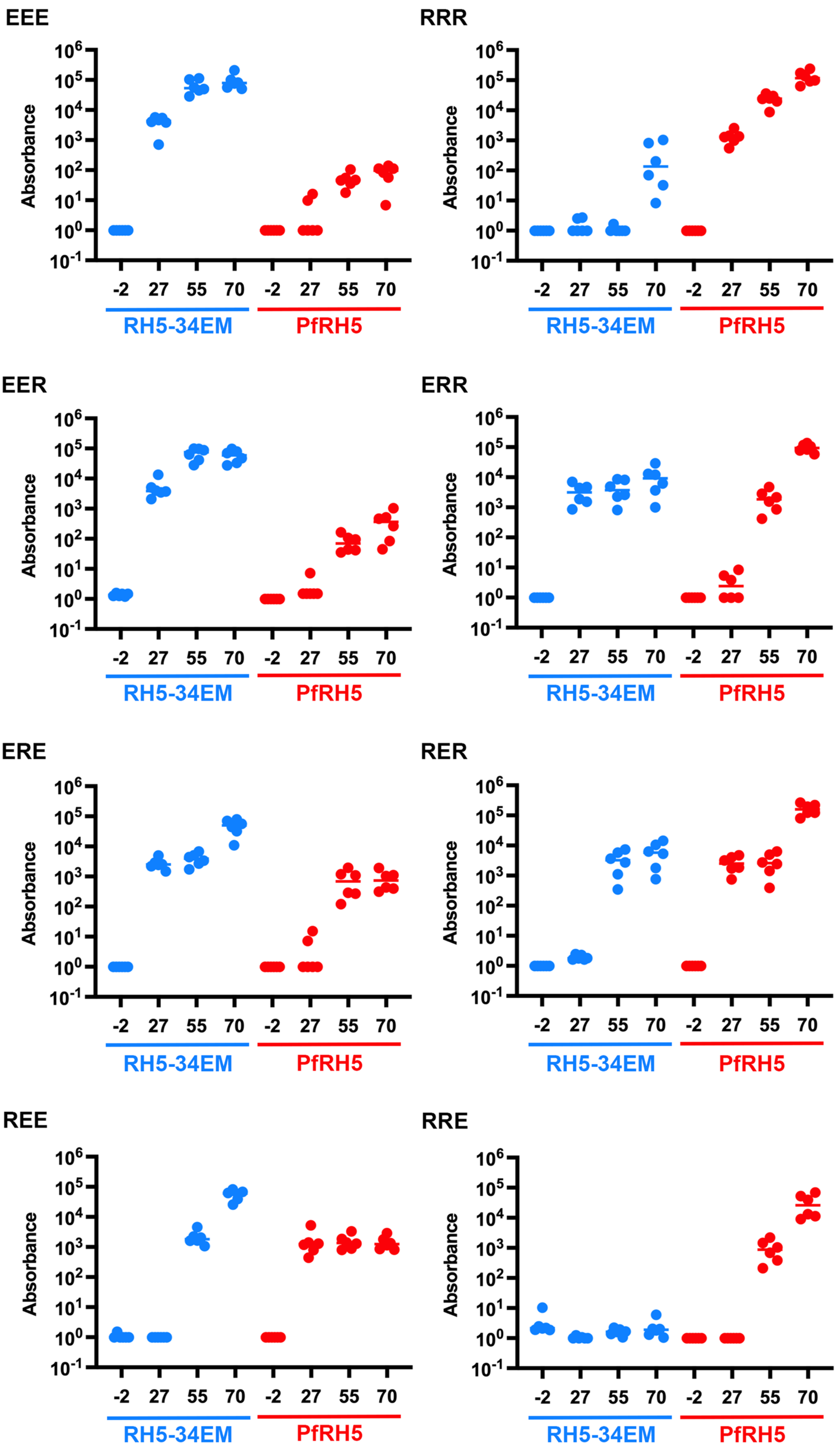
EEE
RRR
EER
ERR
ERE
RER
REE
RRE
Absorbance
10^6
10^5
10^4
10^3
10^2
10^1
10^0
10^-1
-2 27 55 70 -2 27 55 70
RH5-34EM
PfRH5

Figure EV4. ELISA data for different time points during immunisation.

Absorbance measurements of sera against RH5-34EM (blue) and PfRH5 (red) at day -2 (before immunisation) and days 27, 55 and 70 (after the first, second and third vaccine doses). The plots show the eight different vaccine regimens. In each cohort we immunised 6 rats and analysed individually and measured absorbance once for each sample.

 © The Author(s)

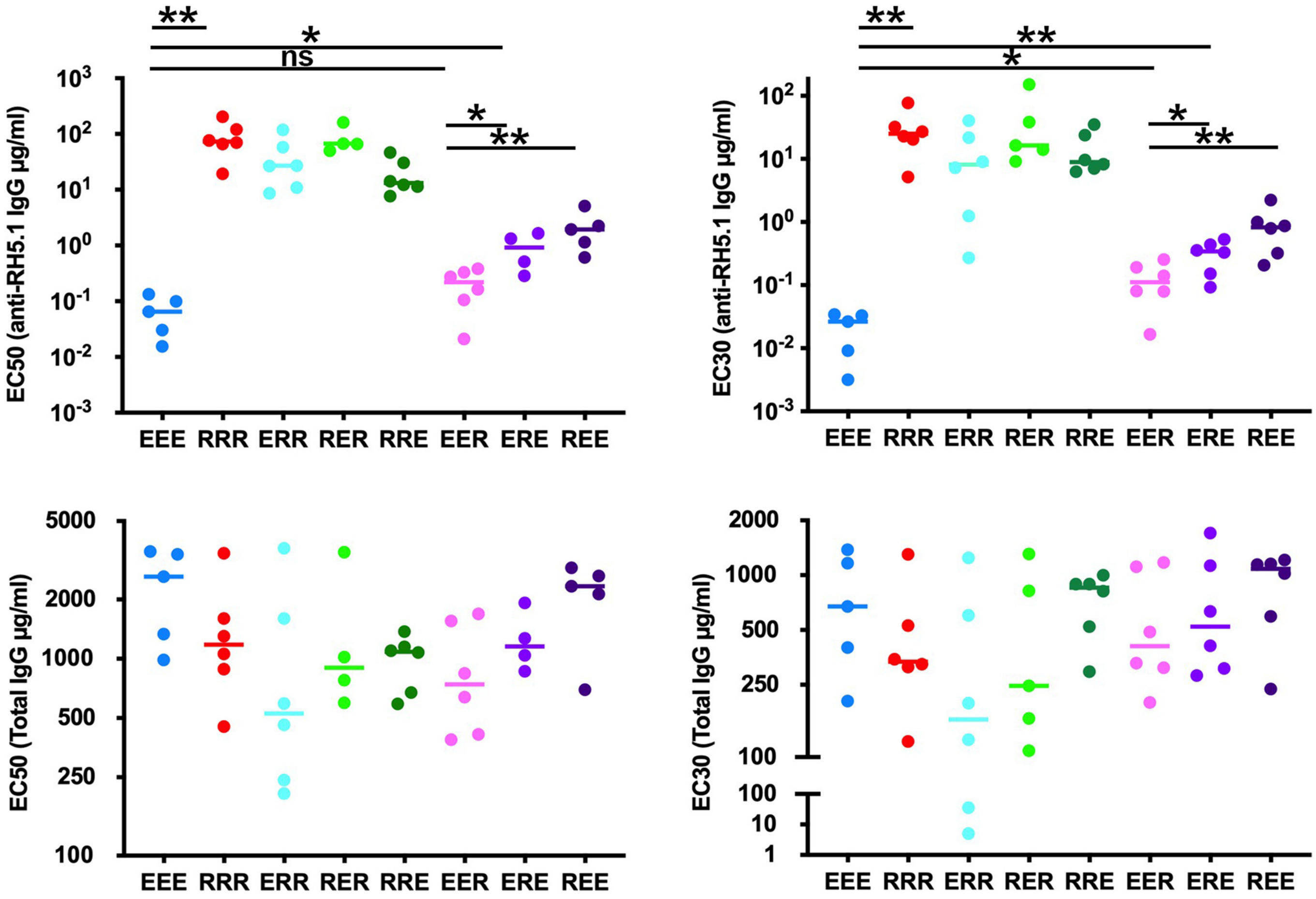

Figure EV5. Comparing EC_{50} and EC_{30}.

Growth-inhibitory activity measurements, as shown in Fig. 5, were analysed to extract EC_{30} and EC_{50} values. The EC_{30} plots here replicate those shown in Fig. 5. In addition, we show plots of EC_{50} for all data points for which growth-inhibitory activity reaches this level. In both cases, we provide data for total IgG and for PfRH5-specific IgG. Statistical significance determined using a two-tailed Mann–Whitney test corrected for multiple comparisons (* indicates <0.05 and ** indicates <0.005). For EC30, for EEE vs RRR, $P = 0.0043$; for EEE vs EER, $P = 0.0303$; for EEE vs ERE, $P = 0.0079$; for EER vs ERE, $P = 0.0411$; for EER vs REE, $P = 0.0043$. For EC50, for EEE vs RRR, $P = 0.0043$; for EEE vs EER, $p = 0.0823$; for EEE vs ERE, $P = 0.0159$; for EER vs ERE, $P = 0.0381$; for EER vs REE, $P = 0.0043$.

© The Author(s)
